# Daily variability in mood and subjective cognitive function: An experience sampling study in young adults

**DOI:** 10.1371/journal.pone.0353474

**Published:** 2026-07-10

**Authors:** Melanie A. Butt, Todd C. Handy

**Affiliations:** Department of Psychology, University of British Columbia, Vancouver, British Columbia, Canada; Polish Academy of Sciences: Polska Akademia Nauk, POLAND

## Abstract

In older adults, greater day-to-day fluctuations in cognitive function appear to be associated with higher rates of cognitive decline, relative to those showing less daily cognitive variability. Such findings suggest that within-person daily deviations from an individual’s typical functioning do not simply represent measurement noise, but rather, vitally inform on cognitive status in older adults. But what about these dynamics in younger adults? Does daily variability in cognition potentially inform on cognitive wellness for those in their neurocognitive prime? Here we addressed this question using an experience sampling method to assess participants’ subjective sense of their cognition and mood three times a day over 14 days in a sample of 215 young, healthy adults. After controlling for four baseline measures of subjective cognition (metacognitive awareness, executive function, propensity for cognitive failures, and memory failures) and four baseline measures of mood (depression, state and trait anxiety, and loneliness), we found that not only did subjectively-assessed cognitive function show significant within-person day-to-day variability across the sample population, but greater person-level cognitive variability across the two-week period predicted lower mean perceived function across the two weeks. While daily cognitive variability also showed a significant correlation with daily measures of both negative and positive mood, notably, some individuals were more resilient to these daily mood impacts on subjective cognition than others. Taken together, our findings suggest that daily variability in subjective cognition and mood may be important predictors for understanding cognitive wellness in younger adult populations.

## Introduction

Day-to-day variability in mood is a hallmark of being human. Indeed, having good and bad days is so commonplace as to be a cliché descriptor of contemporary life. But taken from a cognitive perspective, these normal, natural, and non-clinical shifts in mood are not functionally benign. Rather, accumulating evidence suggests that in otherwise psychologically healthy, young adults, daily fluctuations in mood systematically co-occur with daily fluctuations in cognition as measured via both task performance [1–3] and subjective appraisals of one’s own current cognitive status [4–6]. In turn, this apparent relationship between mood and cognition raises the question driving our study: what might our good days and bad days reveal about our cognition and wellness?

We suggest the question is important for several critical reasons. First, there has been growing awareness that in older adults, greater day-to-day variability in cognitive function may be associated with higher rates of cognitive decline, relative to those older adults showing less daily cognitive variability [7–10]. Such findings have supported the proposal that intraindividual cognitive variability is not simply measurement noise, but rather, has the potential to be diagnostically predictive of cognitive outcomes in older adults [11,12], a conclusion that mirrors what is known about age-related changes in the neural mechanisms supporting cognitive functioning and their impacts on daily cognitive variability [13]. If valid, this expanding understanding of intraindividual variability as a cognitive health predictor in older adults thus demands developing a better understanding of normative, pre-clinical patterns of variability in younger, healthy adults.

Second, from a basic science standpoint, the day itself is a comparatively neglected timescale of cognitive study. For example, changes in cognitive function across the lifespan are widely studied and modeled (e.g., [14,15]). Likewise, there has been interest in understanding if and how cognition might vary with cyclical neurobiological changes across seasons [16–18], gestational periods [19,20], and monthly menstruation cycles [21,22]. At shorter timescales, there has been a recent explosion in studying cognitive fluctuations on the order of seconds to 10s of seconds, including the study of intraindividual variability in trial-by-trial task performance [23–25] and mind wandering (e.g., [26,27]), and across the hours of the day itself [28–30]. Yet despite the 24-hour day being a fundamental time unit at the physical, biological, behavioral, and socio-cultural levels, we have only just begun to gain insight into cognitive variability at this scale of periodicity, one that sits at the juncture of circadian and infradian cycles.

In light of these considerations, we thus designed a study to longitudinally track participants’ self-assessed cognition and mood on a daily basis for a two-week period, controlling for baseline levels of a variety of cognition and mood factors. While the details of our design are described in the methods below, we stress here three central factors that shaped our study protocol. First, while a longer time period of study would have been ideal, we opted for a two-week period to balance length with the expectation that drop-out rates would likely increase logarithmically with study length. Second, we used a validated subjective measure of cognition as a primary outcome variable for two reasons –– (1) we felt a subjective measure was more feasible to administer multiple times a day than a more traditional, task-based or objective measure of cognition, and (2) we wanted to expand on the limited work that we are aware of that used validated measures to assess daily subjective cognitive functioning in young adults [5,31,32]. Finally, in light of questions regarding whether mood and subjective cognitive function are intermixed or highly-intercorrelated constructs [33–35], our study design allowed us to look at their variability over time as a potential way to functionally dissociate between them. That is, to the extent our subjective appraisals of our cognitive function run independent of our good versus bad days as defined by mood, it would suggest that how we feel about how our brains are working is not a simple reflection of mood.

## Method

### Participants

A total of 301 undergraduate and graduate student participants were recruited from the University of British Columbia’s (UBC) Psychology Human Subject Pool and Psychology Paid Participants study list between June 1, 2022 and April 13, 2023. Students received two course credits or $20 Amazon gift cards, respectively, for their participation. A total of 215 students completed the two-week study as described below, and of those, 198 provided at least partial demographic information. The mean reported age was 20.60 (*SD* = 3.75), and included 159 participants identifying as women, 37 identifying as men, and two who identified as non-binary or another gender. Across participants who reported their cultural background, 40.4% (80/198) self-identified as East Asian, 21.7% (43/198) self-identified as European, 12.6% (25/198) self-identified as having multiple backgrounds, 12.1% (24/198) self-identified as South Asian, 7.1% (14/198) self-identified as South East Asian, and 6.1% (12/198) self-identified with other cultural identities. Research was performed in accordance with ethics board guidelines set by UBC’s Behavioural Research Ethics Board, and written informed consent was received from each participant at the outset of the study (Ethics #H22-00495).

### Procedure

The study began by participants coming into the lab for a 30-minute session, where they filled out a set of baseline cognitive and mood measures as described below, and were given instructions on how to complete the daily cognition and mood reports for the subsequent 14 days of assessment. Following these 14 days, the study concluded with participants returning to the lab for a final 30-minute session, which included the same set of baseline cognitive and mood measures as in the first session, as well as a demographics questionnaire. Participants were debriefed upon returning for the second in-lab session at the conclusion of the study. A total of 65 participants completed at least one of the in-lab sessions virtually via Zoom, to comply with provincial COVID-19 safety regulations. The procedure remained identical for these sessions, where the research assistant monitored survey completion and answered all participant questions.

#### Baseline measures –– subjective cognition.

Metacognition was measured using the Metacognitive Awareness Inventory (MAI) [36], which assesses both “knowledge of cognition” and “regulation of cognition”. Executive functioning was measured using the Amsterdam Executive Function Inventory (AEFI) [37], a brief self-reported measure designed specifically for young adults. Propensity for cognitive failures was assessed via the Cognitive Failures Questionnaire (CFQ) [38], which measures self-reported deficits in memory, as well as absent-mindedness and clumsiness. Memory slips were assessed via the Prospective and Retrospective Memory Questionnaire (PRMQ) [39].

#### Baseline measures –– mood.

Depression symptoms were measured using the Center for Epidemiologic Studies Depression Scale (CES-D) [40], where participants rate how often they have felt or behaved a certain way in the past week. Anxiety levels were assessed via the State-Trait Anxiety Inventory (STAI) [41], allowing participants to rate how often they feel a certain way. Loneliness levels were measured using the Revised UCLA Loneliness Scale (UCLA) [42], where participants indicate how often a given statement is descriptive of them.

#### Daily measures.

The PROMIS® Cognitive Function 8a (PROMIS 8a) [43] was used to assess subjective cognition. It is a short-form derived from the PROMIS® Item Bank v2.0 Cognitive Function, assessing momentary subjective experience of cognitive functioning on a 5-point Likert scale, its anchors being “1 (very often)” and “5 (never)”, with greater scores indicating greater perceived cognitive function (e.g., *“I have had trouble forming thoughts”, “It has seemed like my brain was not working as well as usual”*). Question leads and response options were altered to reflect the hour time frame used in this study (e.g., “often (once an hour)” and “very often (several times an hour)”), as compared to the original wording referring to days (“often (about once a day)” and “very often (several times a day)”). The Mood Zoom Questionnaire (MZQ) [44] was used to assess mood states, and includes four items of negative mood (*anxious, sad, angry, irritable*) and two items of positive mood (*energetic, elated*) on a 7-point Likert scale, with anchors of “1 (not at all)” and “7 (very much)”. It was developed as a compact, mobile measure to be used for simple, real-time monitoring of mood states, making it a desirable choice for this study.

#### Daily reporting.

During the two-week assessment period, participants were sent experience sampling surveys three times a day: at 12:00 PM, 5:00 PM, and 9:00 PM. These times were selected to capture their experiences across the day, and asked participants to assess their subjective wellbeing over the past few hours. These mobile questionnaires were hosted on Qualtrics Survey Software and administered via a calendar notification on the participants’ phone, which the researcher set up for them during the first in-lab session. Participants were instructed to fill out the daily questionnaires as soon as possible after receiving the beep, unless the next one was to be delivered sooner, which resulted in some missing data (see *Results* for a description of how these were handled).

#### Additional items.

A further set of measures were included in the participants’ protocols, as part of a second study. These included a baseline personality measure, GPS data collected daily during the 14-day study period, two additional single-item daily mood measures assessing loneliness and stress, and four additional self-report questions collected once daily at 9:00 PM, asking about types of places visited that day (e.g., work, a social engagement), modes of transportation used that day (e.g., walking, transit), types of environments visited that day (e.g., urban, natural), and what portion of that day was spent working or doing school from home (i.e., all, some, or none of the day). These data will not be presented, analyzed, nor detailed methodologically here.

## Results

### Data pre-processing

A total of 301 participants were recruited for the study, while 288 successfully proceeded past the first in-lab session; 13 did not due to technological issues with their smartphones. Prior to data analysis, 73 participants were excluded for the following reasons: fewer than 10 days of recorded survey data (35 participants), scoring 75% or less on the eight attention check questions included during either of the two in-lab sessions (34 participants), and a coding error that made it impossible to distinguish between data sets for some participants (4 total). Thus, the final sample consisted of 215 participants. Data are available on the Open Science Framework (OSF) at https://osf.io/7qbke/.

For participants included in the analysis, two steps were taken to reduce potential biases in the data. First, as part of the study procedure, participants were instructed to complete the daily survey three times a day (at 12:00 PM, 5:00 PM, and 9:00 PM). However, some participants completed multiple surveys within the same time window. As such, when two or more surveys were completed within the approximate time window on a given day, we retained only the first survey. Second, due to the large number of daily questionnaires (42 per participant across the 14 days of the study), participants did not always complete every questionnaire. We thus imputed data for each missing survey by taking the participant’s mean value from the previous two days for the specific time of day for which the datum was missing (12:00 PM, 5:00 PM, or 9:00 PM). This process led to 209 participants having one or more data rows imputed, with a mean of 10.96 rows imputed per participant (median = 9, *SD* = 7.12), ranging from one to 29 imputed rows across the entire sample. Given that we used daily mean values for each participant’s cognition and mood scores, we also assessed missingness at the daily level. Seventy-six participants had at least one day of missing data, with a mean of 1.87 days missing (median = 1, *SD* = 1.04), ranging from one to four days missing (by design, given our exclusion criteria of having at least 10 days’ worth of data).

### Daily associations between subjective cognition and mood

The primary goal of our analyses was to examine daily fluctuations in subjective cognition in young adults, focusing on how this may vary across individuals, as well as identifying how individuals’ fluctuations in daily experience may contribute to cognitive fluctuations. To do so, we estimated a linear mixed-effects model with a random intercept and random slopes, where daily observations of subjective cognition and mood were nested within individuals, and daily positive and negative mood were used to predict daily cognitive states, while baseline scores of subjective cognition and mood acted as control variables. We used person-mean and person-mean centring for daily mood scores, and all variables were scaled to units of standard deviation to ease interpretation. To account for temporal dependency in participants’ daily scores, we used an autoregressive correlation structure to model the assumption that observations closer in time are more similar than those apart. The model was fitted using restricted maximum likelihood estimation. To address observed heteroscedasticity, we applied a participant-level cluster bootstrap with replacement (1000 iterations), including all observations for each resampled participant. Inference was based on studentized bootstrap statistics: *p*-values were computed from the proportion of bootstrap *t*-values exceeding the observed statistic, and 95% confidence intervals were derived from the studentized quantiles. Studentized *t*-statistics are not presented, as they were used only to derive bootstrap *p*-values and confidence intervals. Standard errors were taken as the standard deviation of the bootstrap coefficients, while beta coefficients are reported from the original model. Variance components for the random intercept and slopes are taken from the original model. All significance tests were two-tailed, assessed at an alpha of  .05. All analyses were conducted in R version 4.5.1 [45] using RStudio version 2025.05.1 + 513 [46]. Descriptive statistics for measures of cognition and mood are presented in Table 1, and results of the model are presented in Table 2.

**Table 1:** Descriptive statistics for baseline and daily measures.

	*N*	Min	Max	*M*	*SD*	*Median*
Baseline						
Subjective cognition						
Metacognition (MAI)	413	19	52	38.29	6.40	39
Executive functioning (AEFI)	413	19	35	26.11	2.57	26
Cognitive failures (CFQ)	413	3	90	45.65	14.16	45
Memory (PRMQ)	413	17	79	42.58	10.55	42
Mood						
Depression (CES-D)	413	6	47	19.45	7.58	18
State anxiety (STAI-S)	413	30	60	44.32	5.59	45
Trait anxiety (STAI-T)	413	30	62	45.35	5.96	45
Loneliness (UCLA)	413	26	70	43.83	8.93	44
Daily						
Subjective cognition (PROMIS 8a)	6858	8	40	32.64	6.86	34
Negative mood (MZQ)	6858	1	7	1.91	0.98	1.75
Positive mood (MZQ)	6858	1	7	2.69	1.34	2.5

For baseline measures, *N* represents the number of completed in-lab questionnaires for all participants across both in-lab sessions. For daily measures, *N* represents the number of observations for all participants across the two-week study period.

**Table 2:** Linear mixed-effects model of daily subjective cognition predicted by daily and baseline mood and subjective cognition (imputed data).

						95% CI	
		*β*	*SE*	*DF*	*p*	*Lower*	*Upper*
	Intercept	−0.003	0.03	2783	.894	−0.03	0.03
Daily	Negative mood	**−0.20**	**0.01**	**2783**	**.000**	**−0.22**	**−0.19**
	Positive mood	**0.12**	**0.01**	**2783**	**.000**	**0.11**	**0.14**
	Negative mood – person mean	**−0.36**	**0.04**	**204**	**.000**	**−0.41**	**−0.30**
	Positive mood – person mean	−0.0003	0.03	204	.993	−0.04	0.04
Baseline subjective cognition	Metacognition	0.03	0.04	204	.208	−0.01	0.08
	Executive function	0.03	0.03	204	.209	−0.01	0.06
	Cognitive failures	**−0.10**	**0.06**	**204**	**.030**	**−0.16**	**−0.04**
	Memory failures	**−0.15**	**0.06**	**204**	**.000**	**−0.22**	**−0.09**
Baseline mood	Depression	**−0.19**	**0.05**	**204**	**.000**	**−0.25**	**−0.13**
	State anxiety	**−0.16**	**0.04**	**204**	**.000**	**−0.23**	**−0.10**
	Trait anxiety	**0.08**	**0.05**	**204**	**.026**	**0.02**	**0.16**
	Loneliness	0.002	0.04	204	.942	−0.05	0.06

Model predicted on imputed data. Reported standard errors, *p-*values, and confidence intervals are based on studentized bootstrap estimates, while standardized beta coefficients are from the original model fit.

As a first step, we computed overall model fit, and found that fixed effects alone accounted for 47% of the variance in daily subjective cognition (*R*^2^_m_ = .47), whereas the full model including random effects accounted for 73% of the variance (*R*²_c_ = .73). Next, we separately investigated the amount of variability in daily subjective cognition that could be accounted for by between- and within-subjects’ differences. We found substantial between-person differences in daily subjective cognition across our sample: even after accounting for daily mood as well as baseline subjective cognition and mood as predictors, there exists meaningful variance in daily subjective cognition (τ_0_ = 0.49). Lower daily negative mood strongly predicts daily subjective cognition (*β* = −0.36, 95% CI [−0.41, −0.30], *p* < .001) however this relationship is not significant for positive mood (*β* = −0.0003, 95% CI [−0.04, 0.04], *p* = .993). This indicates that while participants who generally exhibit less negative mood tend to report greater daily perceived cognition, participants who typically exhibit greater positive mood do not tend to have higher daily subjective cognition.

At the within-subjects’ level, we found that when assessing the impacts of negative (τ_1_ = 0.16) and positive (τ_2_ = 0.13) mood separately, each shows moderate variance across individuals in predicting subjective cognition scores. In other words, participants differ moderately in how much daily mood impacts their daily perceived cognition, with the effect of these predictors not being uniform across participants. Examining the average within-person effects, greater negative mood moderately predicted lower daily subjective cognition (*β* = −0.20, 95% CI [−0.22, −0.19], *p* < .001), as did greater positive mood in predicting higher daily subjective cognition (*β* = 0.12, 95% CI [0.11, 0.14], *p* < .001). In other words, on days when participants experience less negative mood than their own average, their perceived cognitive function tends to be higher, and on days when people reported higher positive mood than typical for themselves, their perceived cognitive function was higher as well.

### Cognitive and mood variability as predictors of mean cognition

A secondary goal of our analyses was to examine whether overall, person-level variability in subjective cognition and mood predicts mean subjective cognition scores across the two-week period. To this end, we estimated a linear regression model in which the standard deviations of subjective cognition and mood across the two-week period were used to predict participants’ overall mean perceived cognitive function, while controlling for baseline measures of cognition and mood. Confidence intervals were bootstrapped. We found a moderate predictive relationship between overall subjective cognition score and person-level variability in subjective cognition and mood scores across the two-week period (*F*(11,203) = 25.62, *p* < .001, *R*^*2*^_*adj*_ = .56). Greater cognitive variability was associated with lower mean perceived cognitive function, (*β* = −0.30, 95% CI [−0.41, −0.19], *t*(203) = −5.65, *p* < .001), whereas grea*t*er positive mood variability predicted higher subjective cognition scores (*β* = 0.14, 95% CI [0.04, 0.25], *t*(203) = 2.65, *p* < .01). Negative mood variabili*t*y was not significantly associated with mean subjective cognition (*β* = −0.06, 95% CI [−0.15, 0.03], *t*(203) = −1.11, *p* = .27).

### Control analyses

#### Multilevel factor structure.

To confirm that subjective cognition and mood represent distinct latent factors in our sample, we conducted a multilevel confirmatory factor analysis (MLCFA), specifying a three-factor model (negative mood, positive mood, subjective cognition) estimated using maximum likelihood. The model fit the data well, based on established cutoff criteria [47] (CFI = .951, RMSEA = .041 (90% CI [.040−.043]), SRMR-within =  .043, SRMR-between =  .075). Factor loadings were strong and significant at both levels (see S1 Table). The positive mood factor showed instability at the between-person level (non-significant loadings, one out-of-range estimate), likely reflecting a structural limitation of being just-identified with two indicators, rather than due to insufficient between-person variance, given comparable intraclass correlations across all mood items (.32−.44). Within-person factor correlations were moderate and in the expected direction (negative mood: *r* = −.41, *p* < .0001; positive mood: *r* = .37, *p* < .0001). Negative mood showed a strong between-person relationship with subjective cognition (*r* = −.66, *p* < .0001), while the non-significant positive mood correlation (*r* = −.03, *p* = .44) likely reflects factor instability at this level. All correlations fell below the threshold indicative of poor discriminant validity (*r* > .85; [48]), providing evidence that subjective cognition and mood represent separable latent constructs.

To assess whether a common factor could account for this shared variance across mood and cognition items, we additionally fit a bifactor model (estimated using maximum likelihood), which showed excellent model fit (CFI = .982, RMSEA = .028 (90% CI [.026,  .030]), SRMR-within =  .021, SRMR-between =  .042). Both mood and cognition items loaded strongly onto the common factor at the within- and between-person levels, while positive mood items continued to show instability at the between-person level (see S2 Table). After accounting for the common factor, mood items loaded strongly onto their respective factors at both levels, while several cognition items showed near-zero and insignificant loadings onto the cognition factor. The factor correlations in the bifactor model were substantially lower than those in the three-factor model at both the within-person (negative mood: *r* = −.11, *p* < .05, positive mood: *r* = −.005, *p* = .87) and between-person levels (negative mood: *r* = .13, *p* = .19, positive mood: *r* = −.14, *p* = .15), indicating the common factor captured a substantial portion of shared mood-cognition variance. Taken together, the results from these two models suggest that while subjective cognition and mood share substantial variance, they remain empirically distinguishable constructs.

#### Temporal precedence.

To test whether previous day mood predicted present day perceived cognitive function above and beyond concurrent mood, we fit a time-lagged linear mixed-effects model with the same fixed effects as in our primary model, with the addition of previous day negative and positive mood as within-person lagged predictors. A random intercepts-only structure was specified due to non-convergence of the full random slopes model, and the autoregressive correlation structure was omitted as temporal dependency was explicitly modelled by the lagged predictors. After controlling for prior day perceived cognitive function, previous day positive mood showed minimal prediction of present-day subjective cognition (*β* = −0.03, 95% CI [−0.05, −0.02], *p* < .001), while previous day negative mood showed no significant lagged effect (*β* = 0.02, 95% CI [−0.002, 0.04], *p* = .09). These results suggest that previous day mood does not substantively carry over to influence subjective cognitive function the following day, after controlling for concurrent mood.

#### Moderator analysis.

To examine potential sources of individual differences in daily mood-cognition coupling, we conducted a moderator analysis testing whether baseline subjective cognition and mood traits moderated the within-person effects of daily mood on daily subjective cognition. Linear mixed-effects models were fit similarly to our primary analyses, estimated using restricted maximum likelihood. We first fit a series of separate models, each testing the interactions between one of our eight baseline measures and the within-person negative and positive mood variables. Of the eight traits tested, only metacognitive awareness (MAI) and executive function (AEFI) showed significant interactions with daily mood; all other baseline traits were non-significant moderators. We then fit an omnibus model including both moderators simultaneously. Metacognitive awareness showed significant interactions with both negative (*β* = −0.03, 95% CI [−0.05, −0.01], *p* < .05) and positive mood (*β* = −0.03, 95% CI [−0.05, −0.02], *p* < .01), while executive function showed a significant interaction with negative mood only (*β* = 0.03, 95% CI [0.02, 0.04], *p* < .001). All interaction effects were negligible in magnitude (|*β*| < 0.05), suggesting these traits play a minor role in buffering the influence of daily mood on subjective cognitive function.

#### Additional control analyses.

To verify that we did not systematically introduce a bias in our data by the imputation process described above, we ran the bootstrap model on the raw data set containing complete cases, and compared the results to our imputed model. The estimates for main predictors remained stable across both models, and the amount of variance explained by both models was not meaningfully different. Full results of the complete-case, non-imputed data model are presented in Table 3.

**Table 3:** Linear mixed-effects model of daily subjective cognition predicted by daily and baseline mood and subjective cognition (complete-case data).

						95% CI	
		*β*	*SE*	*DF*	*p*	*Lower*	*Upper*
	Intercept	−0.001	0.03	2667	.954	−0.04	0.03
Daily	Negative mood	**−0.23**	**0.01**	**2667**	**.000**	**−0.25**	**−0.22**
	Positive mood	**0.13**	**0.01**	**2667**	**.000**	**0.12**	**0.15**
	Negative mood – person mean	**−0.33**	**0.04**	**204**	**.000**	**−0.38**	**−0.27**
	Positive mood – person mean	−0.01	0.03	204	.565	−0.05	0.02
Baseline subjective cognition	Metacognition	0.04	0.04	204	.210	−0.01	0.09
	Executive function	0.03	0.03	204	.195	−0.004	0.06
	Cognitive failures	**−0.12**	**0.06**	**204**	**.007**	**−0.19**	**−0.05**
	Memory failures	**−0.13**	**0.06**	**204**	**.006**	**−0.20**	**−0.06**
Baseline mood	Depression	**−0.19**	**0.04**	**204**	**.000**	**−0.24**	**−0.14**
	State anxiety	**−0.15**	**0.05**	**204**	**.000**	**−0.21**	**−0.09**
	Trait anxiety	**0.08**	**0.05**	**204**	**.033**	**0.01**	**0.15**
	Loneliness	0.01	0.04	204	.787	−0.04	0.06

Model predicted on complete-case data. Reported standard errors, *p-*values, and confidence intervals are based on studentized bootstrap estimates, while standardized beta coefficients are from the original model fit.

Additionally, since 8% of participants (17/215) did not complete the demographics questionnaire, we opted out of including demographic variables such as gender in our original model, to preserve a larger sample size. To ensure that excluding gender did not bias our results, we ran an additional model including gender as a predictor variable and compared it to results of our original model but with the reduced sample size (*N* = 198). Both models were refit using maximum likelihood estimation to allow for direct comparison. Model comparison via likelihood ratio test showed no significant improvement with including gender (*χ*^2^(1) = 2.08, *p* = .15), and Akaike Information Criterion and Bayesian Information Criterion values were nearly identical across both models.

To assess the degree of multicollinearity that may be present in our model, Pearson correlations were computed across all variables; no major violations of normality were observed in the Level 1 residuals, and random effects were approximately normally distributed. All significance tests were two-tailed, assessed at an alpha of  .05. As expected, the daily subjective cognition and mood scores were often strongly and significantly correlated with their associated baseline measures. Across domains, daily subjective cognition was moderately associated with baseline measures of mood, as were measures of daily mood with baselines measures of subjective cognition, in the expected directions. Given the large amount of correlation across our predictor and control variables, we suspected multicollinearity and overfitting may be affecting our model. Variance inflation factors (VIF) were calculated between all independent variables; those for cognitive failures (VIF = 4.67), memory failures (VIF = 4.13), depression (VIF = 2.90), and trait anxiety (VIF = 2.06) had values exceeding 2, indicating potential multicollinearity, however no values exceeded the commonly used threshold of 5 [49]. To ensure the robustness of our results, we estimated a reduced model, where only person-mean and person-mean centred values of daily negative and positive mood were used to predict daily subjective cognition. Both models were refit using maximum likelihood estimation to allow for direct comparison. A likelihood ratio test indicated a statistically significant improvement in fit when including the added predictors as compared to the reduced model (*χ*²(8) = 71.82, *p* < .001), therefore we retain the full model.

## Discussion

The goal of our study was to examine the extent to which self-assessed cognition and mood might vary on a daily basis in young, healthy adults. After controlling for four baseline measures of subjective cognition (metacognitive awareness, executive function, propensity for cognitive failures, and memory failures) and four baseline measures of mood (depression, state and trait anxiety, and loneliness), we analyzed the data using both a linear mixed-effects model and a linear regression model. We had two findings of note. First, not only did participants show significant within-person daily deviations in perceived cognitive function, but at the individual level, greater daily cognitive variability was associated with lower overall mean perceived function as averaged across the two-week study window. Second, while daily subjective cognition also showed a significant correlation with daily measures of both negative and positive mood, there was also significant between-individual variability in the strength of this relationship ––where some individuals manifest a strong yoking between subjective cognition and mood, others did not. Taken together, these findings suggest that daily variability in cognition and mood may warrant attention as possible indicators of cognitive wellness in younger adult populations. Given this conclusion, several key points of discussion and clarification follow.

First, it is worth emphasizing that the term ‘variability’ carries two related but distinct meanings across our analyses. In the primary mixed-effects model, variability refers to within-person daily deviations of subjective cognition or mood from an individual’s typical level, which captures a dynamic, day-to-day process. In the secondary analyses, variability is operationalized as the standard deviation of each person’s scores across the two-week period, serving as a trait-like indicator of overall fluctuation. Although both are derived from the same repeated daily measurements, they address distinct questions: the primary analyses examine whether deviations from one’s typical mood level predict concurrent self-reported cognitive states, while the secondary analyses characterize individuals by their degree of overall variability and whether this predicts differences in mean subjective cognition. Given the observational nature of this study design, the directionality of these relationships cannot be established; these findings are best understood as addressing the questions motivating this work, namely the extent to which mood fluctuations and overall variability predict subjective cognitive functioning.

Turning to the person-level characterization of variability, while increased daily cognitive variability in older populations is known to be predictive of future cognitive decline [11,13], our data here raise the possibility that in younger adults, daily variability in subjective cognition may be predictive of a more temporally-immediate or shorter-term cognitive status. Specifically, that greater daily cognitive variability predicated a lower mean perceived cognitive score suggests that in our university sample, the variability effect was being driven by *decreases* in self-assessed cognitive performance, relative to the sample average or norm. Or put alternatively, those showing less overall variability tended to have a higher overall mean level of self-assessed cognitive performance. However, given the population we sampled, this is perhaps not too surprising in hindsight –– not only were they primarily young adults at the developmental apex of their neurocognitive abilities, they were also self-selected as university students. Thus, for a population at or near what might be called a cognitive ceiling, the primary place to go in terms of manifesting variability is *down*. But this detail notwithstanding, our data nevertheless suggest that significant day-to-day variability in perceived cognitive function may be a normal facet of daily experience even in populations considered neurocognitively healthy, and may help to inform on one’s cognitive status.

Second, a key consideration in interpreting the present findings concerns the relationship between subjective cognition and mood as constructs. Subjective cognitive complaints are consistently associated with affective states such as depression and anxiety [50]; across aging, clinical, and young adult populations, these subjective complaints often do not track objective, task-based cognitive performance [51–53]. Thus, some have argued that self-reported cognitive difficulties may more aptly reflect mood disturbance rather than objective cognitive change [54,55]. We note, however, that subjective cognitive functioning is neither reducible to mood nor equivalent to objective cognitive performance; rather, it is a conceptually distinct construct reflecting one’s awareness or appraisal of their cognitive functioning [56]. This distinction matters for interpreting our findings: in a recent meta-analysis by Van Patten et al. [54], the authors concluded that subjective cognition captures appraisal of everyday cognitive functioning across changing day-to-day situations, distinguishable from optimized objective ability measured in controlled laboratory settings. Indeed, our findings show that these appraisals are dynamic, varying reliably within persons across days, and shown to be influenced by mood states. Still, further work is needed to clarify how this variability may relate to broader functional outcomes, and to understand why individuals differ in the degree to which subjective cognition is coupled with mood at the daily level, questions that the present study was not designed to answer. Nevertheless, the overlap of these constructs is itself informative, pointing to the role that mood plays in shaping how we experience and appraise our own cognitive functioning.

Consistent with this distinction, our multilevel factor analyses confirmed empirical separability between subjective cognition and mood, while also revealing considerable overlap. Specifically, the substantial reduction in mood-cognition correlations once a common factor was introduced reflects this shared variance, though we interpret this with caution given the known tendency for bifactor models to demonstrate superior fit due to additional parametrizations [57]. Still, we recognize that some portion of this shared variance likely reflects measurement adjacency rather than true construct covariance [58], a consequence of administering all questionnaires within the same experience sampling prompt to capture contemporaneous processes, and that the unidirectional response options may contribute to acquiescence bias. Nevertheless, our modelling approach allows us to suggest that while the correlated factors solution likely overestimates the true mood-cognition coupling by including shared measurement occasion variance, the common factor solution likely underestimates it by absorbing genuine construct covariance alongside measurement artifact. Further, the multilevel decomposition partials out stable between-person tendencies, which would include a response style such as acquiescence bias, providing a cleaner estimate of the momentary coupling at the within-person level. Thus, it is best to consider the within-person estimates from each model in tandem. Future research may employ similar analytic approaches, as well as include reverse-keyed items and separate measurement occasions, to help clarify the relative contributions of these sources of shared variance.

Third, with respect to the possible role of mood in mediating subjective cognitive states, negative experiences and moods tend to exert a stronger influence on cognition and behaviour than positive ones [59,60]. Importantly, from a validation standpoint, our findings align with this extensive literature, showing that daily negative mood plays a more influential role in shaping cognitive experiences than positive mood. Specifically, we found that participants who, on average, experienced higher levels of negative mood tended to report lower daily perceived cognitive functioning. In contrast, no such relationship was observed for positive mood. Although both positive and negative mood contributed significantly to within-person fluctuations in daily subjective cognition, the effects of negative mood were notably stronger. These data patterns are consistent with research on depression and rumination, which has shown that negative mood states can impair cognitive functioning by promoting repetitive, self-focused thought patterns, leading to increased cognitive load [61–63]. Such effects extend to subjective cognition as well, with negative mood states shown to bias self-appraisal of cognitive performance and propensity for cognitive failures in daily life [35,64]. These findings highlight the need to prioritize the management of negative affect in efforts to support daily cognitive health, even among non-clinical populations.

Finally, variability in daily cognitive functioning may reflect underlying individual differences in psychological resilience or affective control [65,66]. On average, participants reported lower perceived cognitive functioning on days when they experienced higher-than-usual negative mood or lower-than-usual positive mood. However, participants differed meaningfully in how strongly daily mood fluctuations influenced their perceptions of cognitive functioning, suggesting variation in emotional reactivity and cognitive resilience. These within-person patterns suggest that people vary not only in how mood shifts affect their subjective cognition, but also in their perceived capacity to maintain cognitive performance in the face of emotional changes. Of the baseline traits examined as potential moderators, only metacognitive awareness and executive function showed significant interactions with mood beyond the main effects, though these were negligible in magnitude. While the broader emotion regulation literature points to these processes as key in modulating affective influences on cognition [67,68], future work would be needed to establish whether and how these regulatory capacities shape this relationship more directly. Additionally, time-lagged analyses indicated that this coupling occurs concurrently with no spillover to the following day, and that same-day mood remained the strongest predictor of perceived cognitive states, providing some temporal specificity to the observed relationship.

Three specific limitations should be noted to temper interpretation of these findings. First, to prioritize examining the effects of daily mood on subjective cognition, we did not account for other dynamic, daily factors known to impact cognition, such as sleep quality and duration [69], physical activity [70], and acute stress [71]. Although both negative and positive mood explained a meaningful proportion of variance in daily self-reported cognition, additional lifestyle factors likely also contribute to daily cognitive fluctuations. Second, the daily mood questionnaire used in this study included more items assessing negative than positive mood, which may have inadvertently biased participants toward a more negative frame of mind. While the Mood Zoom Questionnaire [44] is validated for ecological momentary assessment protocols and draws on established measures of depression, anxiety, and quality of life, it was originally developed for individuals with bipolar disorder. As such, its items may not be entirely suitable for a broader assessment of emotional experiences in non-clinical populations, given it does not include many items related to positive or neutral affect. Finally, our measure of daily subjective cognition represents an overall self-assessment of one’s cognitive state, and does not include marked breakdowns of facets of cognition, such as memory, attention, or executive function. While individual items relate to these aspects, the composite score offers only a general sense of perceived cognitive functioning. This limits our ability to draw more detailed conclusions about how mood or other daily variables may differentially impact distinct facets of subjective cognition. Future studies would benefit from incorporating more granular assessments that capture domain-specific cognitive variation in everyday life.

## Supporting information

S1 Table Standardized factor loadings and factor correlations from the multilevel confirmatory factor analysis for the three-factor model. (DOCX)

S2 Table Standardized factor loadings and factor correlations from the multilevel confirmatory factor analysis for the common factor model. (DOCX)

## References

[1] Brose A, Schmiedek F, Lövdén M, Lindenberger U. Daily variability in working memory is coupled with negative affect: the role of attention and motivation. Emotion. 2012;12(3):605–17. doi: 10.1037/a0024436 21787075

[2] Nahum M, Sinvani R-T, Afek A, Ben Avraham R, Jordan JT, Ben Shachar MS, et al. Inhibitory control and mood in relation to psychological resilience: an ecological momentary assessment study. Sci Rep. 2023;13(1):13151. doi: 10.1038/s41598-023-40242-1 37573400 PMC10423230

[3] Riediger M, Wrzus C, Schmiedek F, Wagner GG, Lindenberger U. Is seeking bad mood cognitively demanding? Contra-hedonic orientation and working-memory capacity in everyday life. Emotion. 2011;11(3):656–65. doi: 10.1037/a0022756 21534659

[4] Nexha A, Pilz LK, Oliveira MAB, Xavier NB, Borges RB, Frey BN, et al. Greater within- and between-day instability is associated with worse anxiety and depression symptoms. J Affect Disord. 2024;356:215–23. doi: 10.1016/j.jad.2024.04.014 38582128

[5] Guevarra YA, Majeed NM, Hisham EM, Hartanto A. Positive and Negative Affect Differentially Predict Individual Differences and Intra-Individual Changes in Daily Cognitive Failures in Younger and Older Adults. Brain Sci. 2024;14(12):1259. doi: 10.3390/brainsci14121259 39766458 PMC11674940

[6] Aschenbrenner AJ, Jackson JJ. High-frequency assessment of mood, personality, and cognition in healthy younger, healthy older and adults with cognitive impairment. Aging, Neuropsychology, and Cognition. 2024;31(5):914–31. doi: 10.1080/13825585.2023.2284412 39015997 PMC11255411

[7] Dixon RA, Garrett DD, Lentz TL, MacDonald SWS, Strauss E, Hultsch DF. Neurocognitive markers of cognitive impairment: exploring the roles of speed and inconsistency. Neuropsychology. 2007;21(3):381–99. doi: 10.1037/0894-4105.21.3.381 17484601

[8] Gamaldo AA, Allaire JC. Daily fluctuations in everyday cognition: is it meaningful? J Aging Health. 2016;28(5):834–49. doi: 10.1177/0898264315611669 26538267

[9] Bielak AAM, Mogle J, Sliwinski MJ. What Did You Do Today? Variability in Daily Activities is Related to Variability in Daily Cognitive Performance. J Gerontol B Psychol Sci Soc Sci. 2019;74(5):764–71. doi: 10.1093/geronb/gbx145 29240950

[10] Cerino ES, Hooker K, Settersten RA Jr, Odden MC, Stawski RS. Daily linkages among high and low arousal affect and subjective cognitive complaints. Aging Ment Health. 2021;25(5):844–55. doi: 10.1080/13607863.2020.1711863 31933378 PMC7358120

[11] Ferreira D, Machado A, Molina Y, Nieto A, Correia R, Westman E, et al. Cognitive Variability during Middle-Age: Possible Association with Neurodegeneration and Cognitive Reserve. Front Aging Neurosci. 2017;9:188. doi: 10.3389/fnagi.2017.00188 28649200 PMC5465264

[12] Vaughan AC, Birney DP. Within-individual variation in cognitive performance is not noise: why and how cognitive assessments should examine within-person performance. J Intell. 2023;11(6):110. doi: 10.3390/jintelligence11060110 37367512 PMC10302190

[13] MacDonald SWS, Nyberg L, Bäckman L. Intra-individual variability in behavior: links to brain structure, neurotransmission and neuronal activity. Trends Neurosci. 2006;29(8):474–80. doi: 10.1016/j.tins.2006.06.011 16820224

[14] Baltes PB. On the incomplete architecture of human ontogeny. Selection, optimization, and compensation as foundation of developmental theory. Am Psychol. 1997;52(4):366–80. doi: 10.1037//0003-066x.52.4.366 9109347

[15] Baltes PB, Singer T. Plasticity and the ageing mind: an exemplar of the bio-cultural orchestration of brain and behaviour. European Review. 2001;9(1):59–76. doi: 10.1017/S1062798701000060

[16] Meyer C, Muto V, Jaspar M, Kussé C, Lambot E, Chellappa SL, et al. Seasonality in human cognitive brain responses. Proc Natl Acad Sci U S A. 2016;113(11):3066–71. doi: 10.1073/pnas.1518129113 26858432 PMC4801294

[17] Lim ASP, Gaiteri C, Yu L, Sohail S, Swardfager W, Tasaki S, et al. Seasonal plasticity of cognition and related biological measures in adults with and without Alzheimer disease: Analysis of multiple cohorts. PLoS Med. 2018;15(9):e1002647. doi: 10.1371/journal.pmed.1002647 30180184 PMC6122787

[18] Keller MC, Fredrickson BL, Ybarra O, Côté S, Johnson K, Mikels J, et al. A warm heart and a clear head. The contingent effects of weather on mood and cognition. Psychol Sci. 2005;16(9):724–31. doi: 10.1111/j.1467-9280.2005.01602.x 16137259

[19] Hoekzema E, Barba-Müller E, Pozzobon C, Picado M, Lucco F, García-García D, et al. Pregnancy leads to long-lasting changes in human brain structure. Nat Neurosci. 2017;20(2):287–96. doi: 10.1038/nn.4458 27991897

[20] Workman JL, Barha CK, Galea LAM. Endocrine substrates of cognitive and affective changes during pregnancy and postpartum. Behav Neurosci. 2012;126(1):54–72. doi: 10.1037/a0025538 21967374

[21] Protopopescu X, Butler T, Pan H, Root J, Altemus M, Polanecsky M, et al. Hippocampal structural changes across the menstrual cycle. Hippocampus. 2008;18(10):985–8. doi: 10.1002/hipo.20468 18767068

[22] Toffoletto S, Lanzenberger R, Gingnell M, Sundström-Poromaa I, Comasco E. Emotional and cognitive functional imaging of estrogen and progesterone effects in the female human brain: a systematic review. Psychoneuroendocrinology. 2014;50:28–52. doi: 10.1016/j.psyneuen.2014.07.025 25222701

[23] MacDonald SWS, Li S-C, Bäckman L. Neural underpinnings of within-person variability in cognitive functioning. Psychol Aging. 2009;24(4):792–808. doi: 10.1037/a0017798 20025396

[24] Hultsch DF, MacDonald SWS, Dixon RA. Variability in reaction time performance of younger and older adults. J Gerontol B Psychol Sci Soc Sci. 2002;57(2):P101-15. doi: 10.1093/geronb/57.2.p101 11867658

[25] Cole VT, Weinberger DR, Dickinson D. Intra-individual variability across neuropsychological tasks in schizophrenia: a comparison of patients, their siblings, and healthy controls. Schizophr Res. 2011;129(1):91–3. doi: 10.1016/j.schres.2011.03.007 21470829 PMC3771365

[26] Schooler JW, Smallwood J, Christoff K, Handy TC, Reichle ED, Sayette MA. Meta-awareness, perceptual decoupling and the wandering mind. Trends Cogn Sci. 2011;15(7):319–26. doi: 10.1016/j.tics.2011.05.006 21684189

[27] McVay JC, Kane MJ. Drifting from slow to “D’oh!”: working memory capacity and mind wandering predict extreme reaction times and executive control errors. J Exp Psychol Learn Mem Cogn. 2012;38(3):525–49. doi: 10.1037/a0025896 22004270 PMC3395723

[28] Sliwinski MJ, Mogle JA, Hyun J, Munoz E, Smyth JM, Lipton RB. Reliability and Validity of Ambulatory Cognitive Assessments. Assessment. 2018;25(1):14–30. doi: 10.1177/1073191116643164 27084835 PMC5690878

[29] Monk TH, Buysse DJ, Reynolds CF 3rd, Berga SL, Jarrett DB, Begley AE, et al. Circadian rhythms in human performance and mood under constant conditions. J Sleep Res. 1997;6(1):9–18. doi: 10.1046/j.1365-2869.1997.00023.x 9125694

[30] West R, Murphy KJ, Armilio ML, Craik FIM, Stuss DT. Effects of time of day on age differences in working memory. J Gerontol B Psychol Sci Soc Sci. 2002;57(1):P3-10. doi: 10.1093/geronb/57.1.p3 11773218

[31] Gerstorf D, Siedlecki KL, Tucker-Drob EM, Salthouse TA. Executive dysfunctions across adulthood: measurement properties and correlates of the DEX self-report questionnaire. Aging, Neuropsychology, and Cognition. 2008;15(4):424–45. doi: 10.1080/13825580701640374 18584338 PMC3639383

[32] Carlson SE, Suchy Y, Baron KG, Johnson KT, Williams PG. A daily examination of executive functioning and chronotype in bedtime procrastination. Sleep. 2023;46(8):zsad145. doi: 10.1093/sleep/zsad145 37225142

[33] von Stumm S. Is day-to-day variability in cognitive function coupled with day-to-day variability in affect? Intelligence. 2016;55:1–6. doi: 10.1016/j.intell.2015.12.006

[34] Ben-Dor Cohen M, Maeir A, Eldar E, Nahum M. Everyday Cognitive Control and Emotion Dysregulation in Young Adults With and Without ADHD: An Ecological Momentary Assessment Study. J Atten Disord. 2023;27(5):539–53. doi: 10.1177/10870547231153934 36779529 PMC9978869

[35] Csábi E, Kovács FM, Volosin M. Predictors of subjective memory functioning in young adults. BMC Psychiatry. 2025;25(1):155. doi: 10.1186/s12888-025-06568-y 39972297 PMC11841223

[36] Schraw G, Dennison RS. Assessing metacognitive awareness. Contemporary Educational Psychology. 1994;19:460–75. doi: 10.1006/ceps.1994.1033

[37] Van der Elst W, Ouwehand C, van der Werf G, Kuyper H, Lee N, Jolles J. The Amsterdam Executive Function Inventory (AEFI): psychometric properties and demographically corrected normative data for adolescents aged between 15 and 18 years. J Clin Exp Neuropsychol. 2012;34(2):160–71. doi: 10.1080/13803395.2011.625353 22111557

[38] Broadbent DE, Cooper PF, FitzGerald P, Parkes KR. The Cognitive Failures Questionnaire (CFQ) and its correlates. Br J Clin Psychol. 1982;21(1):1–16. doi: 10.1111/j.2044-8260.1982.tb01421.x 7126941

[39] Smith G, Del Sala S, Logie RH, Maylor EA. Prospective and retrospective memory in normal ageing and dementia: a questionnaire study. Memory. 2000;8(5):311–21. doi: 10.1080/09658210050117735 11045239

[40] Radloff LS. The CES-D Scale: A Self-Report Depression Scale for Research in the General Population. Applied Psychological Measurement. 1977;1(3):385–401. doi: 10.1177/014662167700100306

[41] Spielberger CD. Manual for the state-trait anxiety inventory. Consulting Psychologist. 1970.

[42] Russell DW. UCLA Loneliness Scale (Version 3): Reliability, Validity, and Factor Structure. Journal of Personality Assessment. 1996;66(1):20–40. doi: 10.1207/s15327752jpa6601_2 8576833

[43] Cella D, Yount S, Rothrock N, Gershon R, Cook K, Reeve B, et al. The Patient-Reported Outcomes Measurement Information System (PROMIS): progress of an NIH Roadmap cooperative group during its first two years. Med Care. 2007;45(5 Suppl 1):S3–11. doi: 10.1097/01.mlr.0000258615.42478.55 17443116 PMC2829758

[44] Tsanas A, Saunders KEA, Bilderbeck AC, Palmius N, Osipov M, Clifford GD, et al. Daily longitudinal self-monitoring of mood variability in bipolar disorder and borderline personality disorder. J Affect Disord. 2016;205:225–33. doi: 10.1016/j.jad.2016.06.065 27449555 PMC5296237

[45] R Core Team. R: A language and environment for statistical computing. https://www.R-project.org/ 2025. Accessed 2025 July 14.

[46] Posit PBC. RStudio: Integrated Development Environment for R. https://posit.co/ 2025. Accessed 2025 July 14.

[47] Hu L, Bentler PM. Cutoff criteria for fit indexes in covariance structure analysis: Conventional criteria versus new alternatives. Structural Equation Modeling. 1999;6(1):1–55. doi: 10.1080/10705519909540118

[48] Kline RB. Principles and practice of structural equation modeling. 4th ed. New York, NY, US: The Guilford Press. 2016.

[49] Cohen J, Cohen P, West SG, Aiken LS. Applied multiple regression/correlation analysis for the behavioral sciences. 3rd ed. Mahwah, NJ, US: Lawrence Erlbaum Associates Publishers. 2003.

[50] Hill NL, Mogle J, Wion R, Munoz E, DePasquale N, Yevchak AM, et al. Subjective Cognitive Impairment and Affective Symptoms: A Systematic Review. Gerontologist. 2016;56(6):e109–27. doi: 10.1093/geront/gnw091 27342440 PMC5181393

[51] Cacciamani F, Bercu A, Bouteloup V, Grasset L, Planche V, Chêne G, et al. Understanding factors associated with the trajectory of subjective cognitive complaints in groups with similar objective cognitive trajectories. Alzheimers Res Ther. 2023;15(1):205. doi: 10.1186/s13195-023-01348-w 37993894 PMC10666380

[52] Crespo-Sanmiguel I, Zapater-Fajarí M, Garrido-Chaves R, Hidalgo V, Salvador A. Subjective memory complaints in young people; their relationship with objective cognitive performance and the role of neuroticism. An Psicol. 2024;40(2):2. doi: 10.6018/analesps.557291

[53] Zhu X, Ding R, Chen X, Wang X, He P, Wang G. Discrepancy between objective and subjective cognition and its association with the trajectory of symptoms and functioning in depressive patients. Psychol Med. 2024;54(4):808–22. doi: 10.1017/S0033291723002556 37921011

[54] Van Patten R, Mulhauser K, Austin TA, Bellone JA, Cotton E, Chan L, et al. The association between subjective and objective cognitive functioning from a transdiagnostic perspective: An umbrella review and meta-analysis. Clin Psychol Rev. 2025;121:102648. doi: 10.1016/j.cpr.2025.102648 40945212

[55] Marino SE, Meador KJ, Loring DW, Okun MS, Fernandez HH, Fessler AJ, et al. Subjective perception of cognition is related to mood and not performance. Epilepsy Behav. 2009;14(3):459–64. doi: 10.1016/j.yebeh.2008.12.007 19130899 PMC2688662

[56] Jessen F, Amariglio RE, van Boxtel M, Breteler M, Ceccaldi M, Chételat G, et al. A conceptual framework for research on subjective cognitive decline in preclinical Alzheimer’s disease. Alzheimers Dement. 2014;10(6):844–52. doi: 10.1016/j.jalz.2014.01.001 24798886 PMC4317324

[57] Murray AL, Johnson W. The limitations of model fit in comparing the bi-factor versus higher-order models of human cognitive ability structure. Intelligence. 2013;41(5):407–22. doi: 10.1016/j.intell.2013.06.004

[58] Podsakoff PM, MacKenzie SB, Lee J-Y, Podsakoff NP. Common method biases in behavioral research: a critical review of the literature and recommended remedies. J Appl Psychol. 2003;88(5):879–903. doi: 10.1037/0021-9010.88.5.879 14516251

[59] Rozin P, Royzman EB. Negativity Bias, Negativity Dominance, and Contagion. Pers Soc Psychol Rev. 2001;5(4):296–320. doi: 10.1207/S15327957PSPR0504_2

[60] Ito TA, Larsen JT, Smith NK, Cacioppo JT. Negative information weighs more heavily on the brain: the negativity bias in evaluative categorizations. J Pers Soc Psychol. 1998;75(4):887–900. doi: 10.1037//0022-3514.75.4.887 9825526

[61] Whitmer AJ, Gotlib IH. An attentional scope model of rumination. Psychol Bull. 2013;139(5):1036–61. doi: 10.1037/a0030923 23244316 PMC3773498

[62] Brinker JK, Campisi M, Gibbs L, Izzard R. Rumination, Mood and Cognitive Performance. Psychology. 2013;4(3):224–31. doi: 10.4236/psych.2013.43a034

[63] Bruning AL, Mallya MM, Lewis-Peacock JA. Rumination burdens the updating of working memory. Atten Percept Psychophys. 2023;85(5):1452–60. doi: 10.3758/s13414-022-02649-2 36653522 PMC11122689

[64] Carrigan N, Barkus E. A systematic review of cognitive failures in daily life: Healthy populations. Neurosci Biobehav Rev. 2016;63:29–42. doi: 10.1016/j.neubiorev.2016.01.010 26835660

[65] Opitz PC, Lee IA, Gross JJ, Urry HL. Fluid cognitive ability is a resource for successful emotion regulation in older and younger adults. Front Psychol. 2014;5:609. doi: 10.3389/fpsyg.2014.00609 24987387 PMC4060296

[66] Schweizer S, Grahn J, Hampshire A, Mobbs D, Dalgleish T. Training the emotional brain: improving affective control through emotional working memory training. J Neurosci. 2013;33(12):5301–11. doi: 10.1523/JNEUROSCI.2593-12.2013 23516294 PMC6704999

[67] Toh WX, Keh JS, Gross JJ, Carstensen LL. The role of executive function in cognitive reappraisal: A meta-analytic review. Emotion. 2024;24(7):1563–81. doi: 10.1037/emo0001373 38869854

[68] Gross JJ. Emotion Regulation: Current Status and Future Prospects. Psychological Inquiry. 2015;26(1):1–26. doi: 10.1080/1047840X.2014.940781

[69] Lowe CJ, Safati A, Hall PA. The neurocognitive consequences of sleep restriction: A meta-analytic review. Neurosci Biobehav Rev. 2017;80:586–604. doi: 10.1016/j.neubiorev.2017.07.010 28757454

[70] Lambourne K, Tomporowski P. The effect of exercise-induced arousal on cognitive task performance: a meta-regression analysis. Brain Res. 2010;1341:12–24. doi: 10.1016/j.brainres.2010.03.091 20381468

[71] Shields GS, Sazma MA, Yonelinas AP. The effects of acute stress on core executive functions: A meta-analysis and comparison with cortisol. Neurosci Biobehav Rev. 2016;68:651–68. doi: 10.1016/j.neubiorev.2016.06.038 27371161 PMC5003767

