# Supplementary material for: Daily variability in mood and subjective cognitive function: An experience sampling study in young adults
Source: PLoS One. 2026 Jul 10;21(7):e0353474. doi: 10.1371/journal.pone.0353474 (PMC13354066; doi:10.1371/journal.pone.0353474)
Supplement: S1 Table — (DOCX) [file pone.0353474.s001.docx]

**S1 Table: Standardized factor loadings and factor correlations from the multilevel confirmatory factor analysis for the three-factor model.**

|  |  | **Within-person** | | | **Between-person** | | |
| --- | --- | --- | --- | --- | --- | --- | --- |
| **Item** | **Statement** | **Neg. mood** | **Pos. mood** | **Subj. cog.** | **Neg. mood** | **Pos. mood** | **Subj. cog.** |
| **Negative mood items** | | | | | | | |
| 1 | Anxious | .499 |  |  | .761 |  |  |
| 3 | Sad | .517 |  |  | .884 |  |  |
| 4 | Angry | .671 |  |  | .828 |  |  |
| 5 | Irritable | .733 |  |  | .837 |  |  |
| **Positive mood items** | | | | | | | |
| 2 | Elated |  | .647 |  |  | 3.795* |  |
| 6 | Energetic |  | .765 |  |  | .161 |  |
| **Subjective cognition items** | | | | | | | |
| PC2r | My thinking has been slow |  |  | .765 |  |  | .961 |
| PC35r | It has seemed like my brain was not working as well as usual |  |  | .782 |  |  | .965 |
| PC36r | I have had to work harder than usual to keep track of what I was doing |  |  | .750 |  |  | .978 |
| PC42r | I have had trouble shifting back and forth between different activities that require thinking |  |  | .702 |  |  | .958 |
| PC8r | I have had trouble concentrating |  |  | .735 |  |  | .915 |
| PC25r | I have had to work really hard to pay attention or I would make a mistake |  |  | .706 |  |  | .967 |
| PC1r | I have had trouble forming thoughts |  |  | .696 |  |  | .948 |
| PC5r | I have had trouble adding or subtracting numbers in my head |  |  | .398 |  |  | .719 |
| **Factor correlations** | | | | | | | |
| Negative mood ~~ positive mood | | -.323 | | | .041 | | |
| Negative mood ~~ subjective cognition | | -.413 | | | -.657 | | |
| Positive mood ~~ subjective cognition | | .374 | | | -.033 | | |

*Loading exceeds 1.0, indicating a Heywood case.
