# Supplementary material for: Daily variability in mood and subjective cognitive function: An experience sampling study in young adults
Source: PLoS One. 2026 Jul 10;21(7):e0353474. doi: 10.1371/journal.pone.0353474 (PMC13354066; doi:10.1371/journal.pone.0353474)
Supplement: S2 Table — (DOCX) [file pone.0353474.s002.docx]

**S2 Table: Standardized factor loadings and factor correlations from the multilevel confirmatory factor analysis for the common factor model.**

|  |  | **Within-person** | | | | **Between-person** | | | |
| --- | --- | --- | --- | --- | --- | --- | --- | --- | --- |
| **Item** | **Statement** | **Neg. mood** | **Pos. mood** | **Subj. cog.** | **Common** | **Neg. mood** | **Pos. mood** | **Subj. cog.** | **Common** |
| **Negative mood items** | | | | | | | | | |
| 1 | Anxious | .355 |  |  | .344 | .320 |  |  | .673 |
| 3 | Sad | .438 |  |  | .245 | .645 |  |  | .567 |
| 4 | Angry | .710 |  |  | .172 | .878 |  |  | .386 |
| 5 | Irritable | .672 |  |  | .286 | .611 |  |  | .576 |
| **Positive mood items** | | | | | | | | | |
| 2 | Elated |  | .863 |  | −.227 |  | .712 |  | .098 |
| 6 | Energetic |  | .490 |  | −.318 |  | .864 |  | −.036 |
| **Subjective cognition items** | | | | | | | | | |
| PC2r | My thinking has been slow |  |  | −.030 | −.813 |  |  | −.330 | −.953 |
| PC35r | It has seemed like my brain was not working as well as usual |  |  | .072 | −.791 |  |  | −.167 | −.958 |
| PC36r | I have had to work harder than usual to keep track of what I was doing |  |  | .385 | −.680 |  |  | .071 | −.988 |
| PC42r | I have had trouble shifting back and forth between different activities that require thinking |  |  | .358 | −.633 |  |  | .062 | −.964 |
| PC8r | I have had trouble concentrating |  |  | .290 | −.683 |  |  | −.024 | −.917 |
| PC25r | I have had to work really hard to pay attention or I would make a mistake |  |  | .320 | −.646 |  |  | .056 | −.971 |
| PC1r | I have had trouble forming thoughts |  |  | .037 | −.711 |  |  | −.164 | −.936 |
| PC5r | I have had trouble adding or subtracting numbers in my head |  |  | .093 | −.384 |  |  | −.084 | −.711 |
| **Residual factor correlations** | | | | | | | | | |
| Negative mood ~~ positive mood | | -.195 | | | | .179 | | | |
| Negative mood ~~ subjective cognition | | -.105 | | | | .127 | | | |
| Positive mood ~~ subjective cognition | | -.005 | | | | -.135 | | | |
